# lncRNA-mRNA expression profiles and functional networks of mesenchymal stromal cells involved in monocyte regulation

**DOI:** 10.1186/s13287-019-1306-x

**Published:** 2019-07-16

**Authors:** Ming Li, Zhongyu Xie, Zhaopeng Cai, Fang Su, Guan Zheng, Jinteng Li, Shan Wang, Shuizhong Cen, Wenjie Liu, Su’an Tang, Guiwen Ye, Zhaofeng Li, Rujia Mi, Yiqian Pan, Peng Wang, Yanfeng Wu, Huiyong Shen

**Affiliations:** 1 Department of Orthopedics, The Eighth Affiliated Hospital, Sun Yat-sen University, No. 3025, Shennan Middle Road, Futian District, Shenzhen, 518033 Guangdong China; 2 Department of Orthopedics, Sun Yat-sen Memorial Hospital, Sun Yat-sen University, Guangzhou, 510120 People’s Republic of China; 3 Guangdong Provincial Key Laboratory of Malignant Tumor Epigenetics and Gene Regulation, Sun Yat-Sen Memorial Hospital, Sun Yat-Sen University, Guangzhou, 510120 People’s Republic of China; 4 Center for Biotherapy, Sun Yat-sen Memorial Hospital, Sun Yat-sen University, 107 Yan Jiang Road West, Guangzhou, 510120 Guangdong China; 5 Department of Orthopedics, Nanfang Hospital, Southern Medical University, Guangzhou, 510120 People’s Republic of China; 6 Zhongshan School of Medicine, Sun Yat-sen University, Guangzhou, 510120 People’s Republic of China

**Keywords:** Mesenchymal stromal cells, Monocytes, Long non-coding RNA, Immunoregulation

## Abstract

**Background:**

The goals of this study were to explore the expression profiles and functional networks of long non-coding RNAs (lncRNAs) and messenger RNAs (mRNAs) in mesenchymal stromal cells (MSCs) involved in regulating the function of monocytes and to clarify the mechanisms by which MSCs exert immunoregulatory effects on monocytes.

**Methods:**

MSCs and CD14+ monocytes were separately isolated. The immunoregulatory effects of MSCs on monocytes were determined by flow cytometry. lncRNAs and mRNAs that were differentially expressed (DE) between the control group (MSCs only) and co-culture group (MSCs co-cultured with monocytes) were identified through high-throughput sequencing and bioinformatic analyses and were confirmed by qRT-PCR. Bioinformatic analyses were performed to identify the critical biological functions and signalling pathways involved in MSC-mediated monocyte regulation and to identify the functional networks formed between DE mRNAs and lncRNAs.

**Results:**

MSCs showed a strong ability to induce monocyte migration but inhibited monocyte differentiation into M1 macrophages. A total of 145 DE lncRNAs and 768 DE mRNAs were identified between the control and co-culture groups. Significant fold changes in lncRNAs and mRNAs were confirmed by qRT-PCR. GO analysis demonstrated that DE mRNAs and lncRNAs were highly associated with terms related to binding and biological regulation. KEGG analysis revealed 122 significantly regulated pathways, including the cytokine-cytokine receptor pathway and chemokine signalling pathway. Interaction and co-expression networks were constructed for DE mRNAs and lncRNAs, and several key microRNAs were identified in the competitive endogenous RNA (ceRNA) network. Target genes of the DE lncRNAs were analysed to predict critical mRNA-lncRNA axes involved in the immunoregulatory function of MSCs.

**Conclusions:**

Our research describes the lncRNA and mRNA expression profiles and functional networks involved in MSC-mediated regulation of monocytes. These results provide possible molecular mechanisms for the immunoregulatory function of MSCs and may help to elucidate possible molecular therapeutic targets in MSCs for the treatment of autoimmune diseases.

**Electronic supplementary material:**

The online version of this article (10.1186/s13287-019-1306-x) contains supplementary material, which is available to authorized users.

## Background

Mesenchymal stromal cells (MSCs) are cells with multiple forms of differentiation potential and self-renewal ability [1]. These cells are derived from the mesoderm and play a role in regulating many types of immune cells [2], including monocytes [3, 4], dendritic cells [5] and T lymphocytes [6]. These powerful features contribute to their critical role in the clinical treatment of various diseases, such as systemic lupus erythaematosus (SLE) [7, 8] and graft-versus-host disease (GVDH) [9, 10]. In addition, according to recent studies, abnormal immunoregulation by MSCs could lead to several autoimmune diseases [11–13]. Therefore, more in-depth studies should be conducted to clarify the specific mechanisms by which MSCs exert immunoregulatory functions. A better understanding of these mechanisms may help to improve the curative effect of MSCs and illuminate the pathogenesis of autoimmune diseases.

Monocytes, which are derived from haematopoietic stem cells, are an important type of immune cell in vivo [14]. These cells develop in the bone marrow, migrate to inflamed tissue and then differentiate into macrophages and dendritic cells [15]. These processes are controlled by many factors that are critical for maintaining homeostasis of the immune system in vivo [16–18]. MSCs can efficiently regulate monocyte migration and differentiation by secreting various cytokines and chemokines [2, 4, 19]. However, the specific immunoregulatory mechanisms by which MSCs control monocytes must be addressed.

Long non-coding RNAs (lncRNAs) are a type of non-protein coding RNA greater than 200 nt in length [20]. lncRNAs are important epigenetic regulators and thus play crucial roles in various cell biology behaviours [21]. Specifically, lncRNAs are widely involved in the regulation of immune system homeostasis [22, 23]. However, it is still unclear whether lncRNAs control the immunoregulatory function of MSCs in immunocytes, particularly in monocytes.

The current study presents an integrative analysis of lncRNA-mRNA expression profiles and functional networks involved in MSC-mediated regulation of monocyte functions. These results improve our understanding of the role of lncRNAs in the immunoregulatory ability of MSCs and could indicate potential targets to improve the curative effect of MSCs.

## Methods

### Cell isolation and culture

MSCs were purified and isolated according to our previously reported methods [24]. Donor information is listed in Additional file 1: Table S1. Briefly, the bone marrow was extracted with a sterile bone needle, centrifuged to obtain the upper layer by density gradient, centrifuged again and then cultured in Dulbecco’s modified Eagle’s medium (DMEM; Gibco, New York, USA) containing 10% foetal bovine serum (FBS; Gibco, New York, USA). The medium was replaced once every 3 days. In all experiments, MSCs at passages 3–5 were used. Molecular markers of MSCs were detected by flow cytometry. MSC osteogenic, adipogenic and chondrogenic differentiation was confirmed by previously reported methods [25]. Peripheral blood mononuclear cells (PBMCs) were isolated by density gradient centrifugation. CD14+ monocytes were isolated from PBMCs using CD14 MicroBeads (Miltenyi Biotec, Bergisch Gladbach, Germany) according to the manufacturer’s protocol.

### Co-culture of MSCs and CD14+ monocytes

MSCs were co-cultured with CD14+ monocytes using Polycarbonate Membrane Transwell® Inserts (Corning, New York, USA). The system includes six-well plates containing inserts with a 0.4-μm pore size. 1 × 10^5^ MSCs were cultured in the base of the wells, and 1 × 10^6^ CD14+ monocytes were seeded in the upper inserts. Both cell types were cultured in RPMI 1640 medium (Gibco, New York, USA) containing 10% FBS at 37 °C in a 5% CO_2_ atmosphere. The 5.0-μm pore Transwell system was used for the migration assay.

### Flow cytometry

For phenotypic analyses, MSCs were centrifuged and then resuspended in phosphate-buffered saline (PBS). Cells were then incubated with human CD29-phycoerythrin (PE), CD34-allophycocyanin (APC), CD44-fluorescein isothiocyanate (FITC), CD45-FITC, CD105-FITC or HLA-DR-PE antibodies for 30 min. To assess the purity of CD14+ monocytes, we incubated cells with the CD14-FITC antibody for 30 min. For macrophage polarization assays, CD14+ monocytes were cultured with or without MSCs for 5 days, incubated with an anti-HLA-DR-PE antibody and then incubated with fixation medium (Invitrogen) for 15 min (Additional file 4). After three washes, the cells were incubated with a permeabilization medium (Invitrogen) plus an anti-CD68 antibody (BD Pharmingen) for 30 min. For migration assays, CD14+ monocytes that migrated into the lower chambers after 12 h of co-culture were collected and counted by flow cytometry. All labelled cells were detected using a BD Influx cell sorter (BD Biosciences). All of the antibodies used for flow cytometry were purchased from BD Biosciences (New York, USA).

### Library construction and high-throughput sequencing

Five MSC samples cultured with CD14+ monocytes (co-culture group; samples B1–B5) and five samples cultured without CD14+ monocytes (control group; samples A1–A5) were separately treated with TRIzol (TAKARA). RNA was collected from each sample according to the manufacturer’s protocol. RNA integrity was evaluated using the Agilent 2200 TapeStation (Agilent Technologies, USA); each sample that had an RIN above 7.0. rRNA was removed using a ribosomal RNA depletion kit, and the RNA was fragmented (average fragment length was approximately 200 nt) and reverse-transcribed into single-stranded cDNA. Then, double-stranded cDNA was synthesized, purified and treated with terminal repair and ligation primers according to the instructions from the NEBNext® Ultra™ RNA Library Prep Kit for Illumina (NEB, USA). After PCR amplification and purification, libraries were paired-end sequenced (PE150, sequencing reads were 150 bp) at Guangzhou RiboBio Co., Ltd. (Guangzhou, China) using the IlluminaHiSeq 3000 platform.

### Real-time quantitative reverse transcription-polymerase chain reaction (qRT-PCR)

Total RNA was isolated from MSCs cultured with or without CD14+ monocytes using TRIzol according to the manufacturer’s protocol. Complementary DNA was transcribed using the PrimeScript RT reagent kit (TaKaRa, Dalian, China). qRT-PCR was then performed, and the results were analysed using the 2^−ΔΔCt^ method. A detailed method can be found in our previous study [25]. The forward and reverse primers for each gene are listed in Additional file 2: Table S2.

### Expression analysis

The raw data were filtered to remove low-quality reads, evaluate sequencing quality and remove ribosomal RNA. The resulting high-quality data were normalized to the expected number of reads per kilobase of transcript sequence per million base pairs sequenced (RPKM) and analysed using the DESeq2 method based on the negative binomial generalized linear model. Genes displaying significant fold changes (log2FoldChange > 1; *q* value< 0.05) between sample groups were considered for additional investigation.

### mRNA and lncRNA annotation and enrichment analysis

GO term enrichment analyses were performed to acquire annotation and enrichment information. Briefly, all genes of the species were selected as background genes, and GO was performed with KOBAS3.0 software, which provides label classification of gene function and gene product attributes (http://www.geneontology.org). *P* values were calculated using the hypergeometric distribution method. *P* < 0.05 was used as the significance threshold to obtain high-frequency annotations with statistical significance relative to controls.

KEGG pathway annotation was used to categorize DE genes by biological pathway. *P* values were calculated using Fisher’s exact test. *P* < 0.05 was used as the threshold for determining statistical significance of signal transduction and disease pathway enrichment. DE mRNAs and enriched pathways were mapped using KEGG pathway annotation with KOBAS3.0 software (http://www.genome.jp/kegg).

### Signal network analysis

Based on the KEGG pathway annotation and mRNA and lncRNA DE results, R was used to generate pathway nets representing the interactions between the pathways associated with DE genes.

### Interaction analysis and co-expression network analysis

We used the natural language processing (NLP) method to perform text mining from the PubMed summary database in order to obtain information about all genes or proteins related to the target object. Based on the above data, we obtained the overall construction of the interaction relationship and then built an mRNA-lncRNA co-expression network using the Cytoscape software. The co-expression network was constructed by calculating the Pearson correlation coefficients and *P*-values between multiple genes. The transcripts were filtered using a COR of > 0.85 and a *P* value of < 0.05.

### ceRNA network analysis

The mRNAs and lncRNAs selected for the co-expression network analysis were the same as those used to predict miRNA targets using the miRbase. The miRNAs obtained through these predictions were screened using the miRanda and TargetScan programmes. lncRNAs and mRNAs possessing microRNA recognition elements (MREs) for the targeted miRNAs were predicted using RNA22. The competitive endogenous RNA (ceRNA) network was constructed and illustrated using Cytoscape (v3.4.0).

### Statistical analysis

Statistical analyses were performed using SPSS 22.0 software (Chicago, IL, USA). All qPCR results are expressed as the mean ± standard deviation (SD). Spearman correlation was used to determine the relationship between lncRNAs and their target genes. *P* values < 0.05 were considered significant.

## Results

### MSC-mediated regulation of CD14^+^ monocytes

**Fig. 1:**
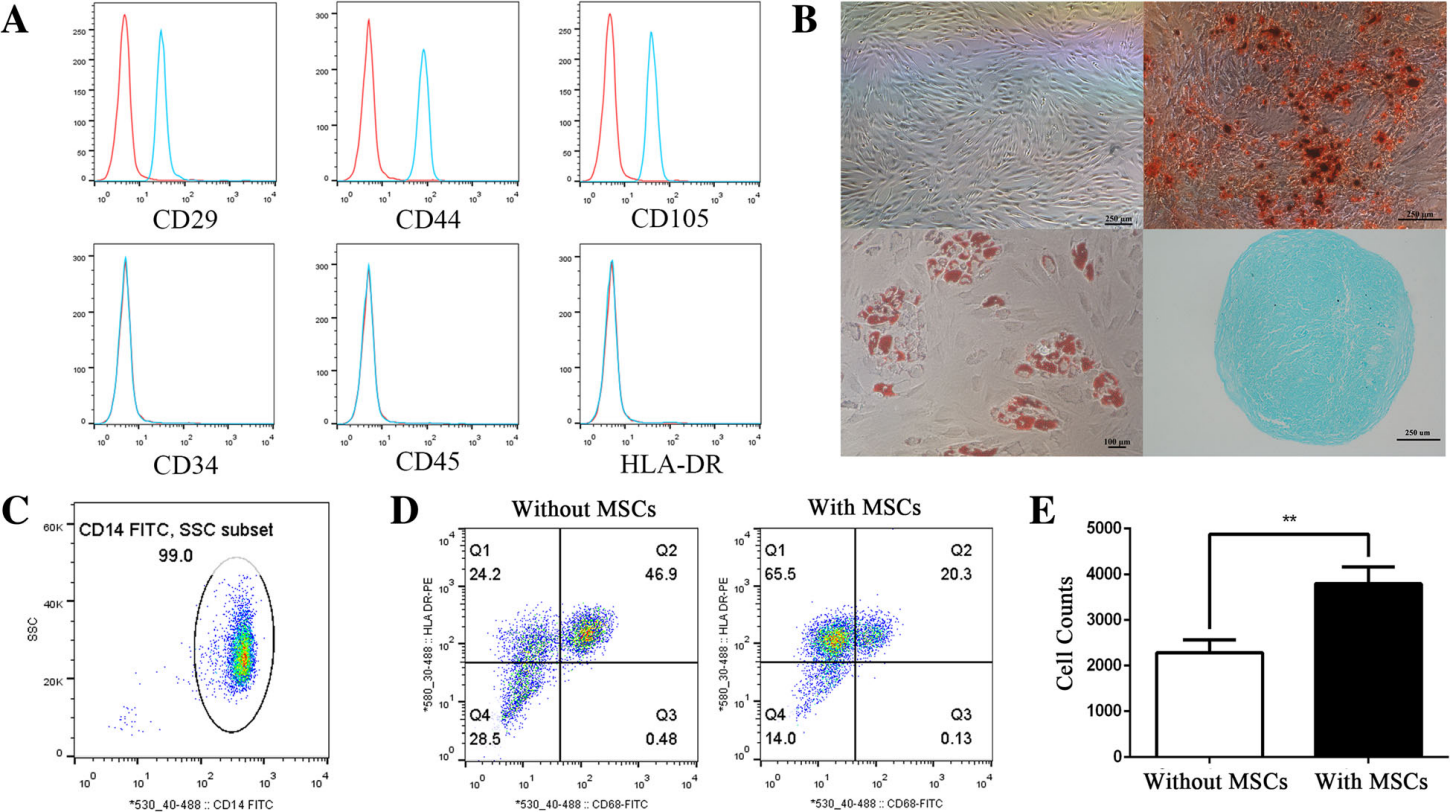
MSCs and their regulatory function in CD14^+^ monocytes. **a** Cell markers of MSCs were analysed by flow cytometry. MSCs were negative for CD45, CD34 and HLA-DR and positive for CD29, CD105 and CD44. **b** Alizarin red S staining (× 40), ALP staining (× 40), oil red staining (× 100) and toluidine blue staining (× 40) were used to detect MSC differentiation. **c** Flow cytometric analysis of the purity of CD14^+^ monocytes. **d** The differentiation ratio of M1 macrophages (CD68 and HLA-DR positive) was decreased upon co-culture with MSCs. **e** MSCs promote monocyte migration, as detected by flow cytometry. Introduction of the RNA-seq experiments: MSCs and CD14+ monocytes were separately isolated. lncRNAs and mRNAs that were DE between the control group (MSCs only) and co-culture group (MSCs co-cultured with monocytes) were identified through high-throughput sequencing and bioinformatic analyses and confirmed by qRT-PCR. Bioinformatic analyses were performed to identify key biological functions and signalling pathways involved in MSC-mediated monocyte regulation. Additionally, functional networks were constructed from the DE mRNAs and lncRNAs

MSCs were isolated and purified as previously described [26]. Flow cytometric analyses demonstrated that all MSCs were positive for CD29, CD44 and CD105 and negative for CD34, CD45 and HLA-DR (Fig. 1a). In addition, MSCs were capable of differentiating into osteoblasts, adipoblasts and chondroblasts (Fig. 1b). The purity of isolated CD14^+^ monocytes was 99.0% (Fig. 1c). Through polarization assays, we demonstrated that the ratio of CD14^+^ monocytes that differentiated into M1 macrophages was significantly lower when these cells were co-cultured with MSCs compared with those cultured without MSCs (Fig. 1d). Additionally, migration assays showed that CD14^+^ monocyte migration was increased upon co-culture with MSCs (Fig. 1e). These results indicated that MSCs potently regulate CD14^+^ monocytes, promoting CD14^+^ monocyte migration but inhibiting M1 macrophage polarization.

### Identification of differentially expressed lncRNAs and mRNAs

**Fig. 2:**
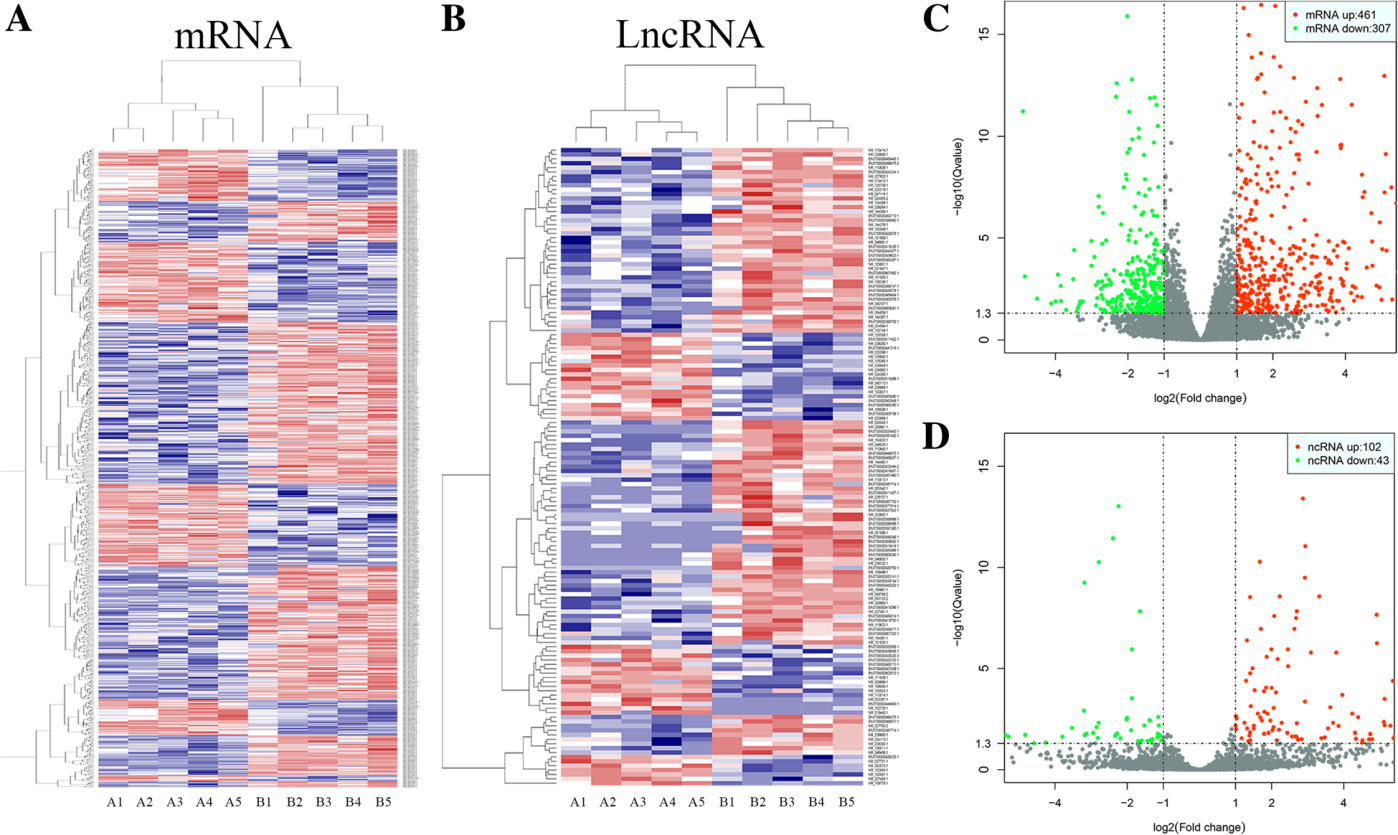
Identification of DE lncRNAs and mRNAs. **a** Heatmaps of DE mRNAs between the control group and the co-culture group. **b** Heatmaps of DE lncRNAs between the control group and the co-culture group. **c** Volcano plots of DE mRNAs between the control group and the co-culture group. **d** Volcano plots of DE lncRNAs between the control group and the co-culture group. In each heatmap, A indicates the control group (only MSCs) and B indicates the co-culture group (MSCs co-cultured with monocytes)

A total of 768 mRNAs were differentially expressed (DE) in MSCs co-cultured with CD14^+^ monocytes compared to MSCs cultured alone. Among these genes, 461 mRNAs were upregulated and 307 mRNAs were downregulated. The DE mRNAs are depicted using a clustergram (Fig. 2a) and volcano plots (Fig. 2c). The 20 mRNAs with the largest fold changes are shown in Table 1. Several cytokines are contained within this list, including CCL8, CXCL6, CCL20, CXCL5, CXCL8 and CXCL3, which are secreted by MSCs and are important for regulating the function of CD14^+^ monocytes. A total of 145 lncRNAs, including 102 upregulated and 43 downregulated lncRNAs, were DE in MSCs co-cultured with CD14^+^ monocytes compared to MSCs cultured alone. The DE lncRNAs are depicted in a clustergram (Fig. 2b) and volcano plots (Fig. 2d). The 10 lncRNAS with the largest fold changes are shown in Table 2.

**Table 1:** The characteristics of mRNAs with the largest fold change

Gene name	Accession no.	Fold change	Regulation
CCL8	NM_005623.2	8.321599761	Up
SAA2	NM_001127380.2	8.097171396	Up
C15orf48	NM_032413.3	7.886234599	Up
CSF2	NM_000758.3	7.702026899	Up
CXCL6	NM_002993.3	7.373507956	Up
CCL20	NM_004591.2	7.36511603	Up
LIPK	NM_001080518.1	7.088336879	Up
CXCL5	NM_002994.4	7.033329067	Up
LIPM	NM_001128215.1	6.863484456	Up
CXCL8	NM_000584.3	6.8596902	Up
STON1-GTF2A1L	NM_172311.2	6.822896276	Up
C8A	NM_000562.2	6.735926684	Up
GIMAP8	NM_175571.3	6.455076221	Up
CXCL3	NM_002090.2	6.391293509	Up
RERG	NM_032918.2	6.377615983	Up
TNIP3	NM_024873.5	6.275240083	Up
DLC1	NM_024767.3	6.104326419	Down
CXCL10	NM_001565.3	6.174218941	Up
PDE4D	NM_001197223.1	5.990349377	Up
IGF1	NM_001111283.2	5.973815259	Up

**Table 2:** The characteristics of lncRNAs with the largest fold change

Accession no.	Fold change	Regulation	Chromosome	Strand	Start	End	Class	Size (bp)
NR_026861	7.578345	Up	6	−	165,924,048	165,988,039	Intronic	1123
ENST00000524942.1	6.514236	Up	11	−	62,077,277	62,082,184	Intergenic	788
ENST00000559869.1	6.127577	Up	15	+	45,448,427	45,461,390	Antisense	487
ENST00000565968.1	5.970342	Up	14	−	59,919,423	59,920,339	Antisense	507
NR_046852.1	5.732526	Up	3	−	171,876,352	171,900,740	Antisense	427
ENST00000411427.1	5.683977	Up	21	−	41,441,056	41,445,708	Antisense	1116
ENST00000551450.1	5.65502	Up	12	+	111,812,793	111,813,420	Sense	569
ENST00000442252.1	5.533016	Up	7	−	22,571,607	22,661,792	Intronic	1282
ENST00000449463.1	5.28627	Down	6	−	78,604,467	78,606,036	Intergenic	387
NR_015440.1	5.37172	Down	1	−	3,059,617	3,067,725	Intergenic	3708

### Validation of DE mRNA and lncRNA expression levels

**Fig. 3:**
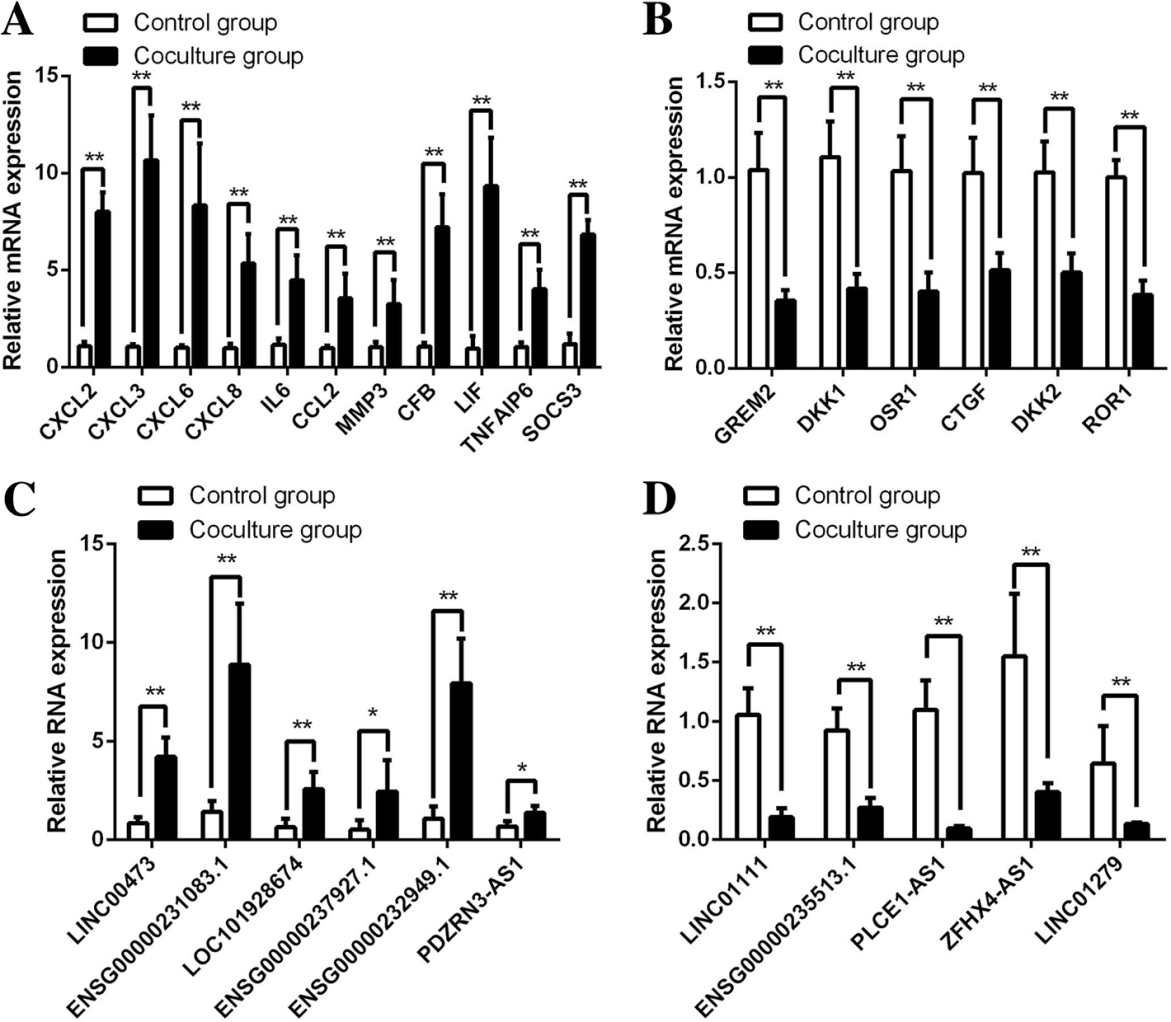
Validation of DE mRNAs and lncRNAs. **a**, **b** DE mRNAs were confirmed by qPCR. **c**, **d** DE lncRNAs were confirmed by qPCR. CXCL2, C-X-C motif chemokine ligand 2; CXCL3, C-X-C motif chemokine ligand 3; CXCL6, C-X-C motif chemokine ligand 6; CXCL8, C-X-C motif chemokine ligand 8; IL6, interleukin 6; CCL2, C-C motif chemokine ligand 2; MMP3, matrix metallopeptidase 3; CFB, complement factor B; LIF, LIF, interleukin 6 family cytokine; TNFAIP6, TNF alpha-induced protein 6; SOCS3, suppressor of cytokine signalling 3; GREM2, gremlin 2, DAN family BMP antagonist; DKK1, dickkopf WNT signalling pathway inhibitor 1; OSR1, odd-skipped related transcription factor 1; CTGF, connective tissue growth factor; DKK2, dickkopf WNT signalling pathway inhibitor 2; ROR1, receptor tyrosine kinase such as orphan receptor 1

To confirm the results of RNA sequencing, several important DE mRNAs and lncRNAs were assessed by qPCR. As shown in Fig. 3a, CXCL2, CXCL3, CXCL6, CXCL8, IL-6, CCL2, MMP3, CFB, LIF, TNFAIP6 and SOCS3 were upregulated in MSCs co-cultured with CD14^+^ monocytes, while GREM2, DKK1, OSR1, CTGF and ROR1 were downregulated (Fig. 3b). Moreover, qPCR results demonstrated that LINC00473, ENSG00000231083.1, LOC101928674, ENSG00000237927.1, ENSG00000232949.1 and PDZNR3-AS1 were upregulated, while LINC01111, ENSG00000235513.1, PLCE1-AS1, ZFHX4-AS1 and LINC01279 were downregulated in MSCs co-cultured with CD14^+^ monocytes (Fig. 3c, d). All qPCR results were consistent with the RNA sequencing results, confirming the reliability of the sequencing data.

### GO and KEGG analysis

**Fig. 4:**
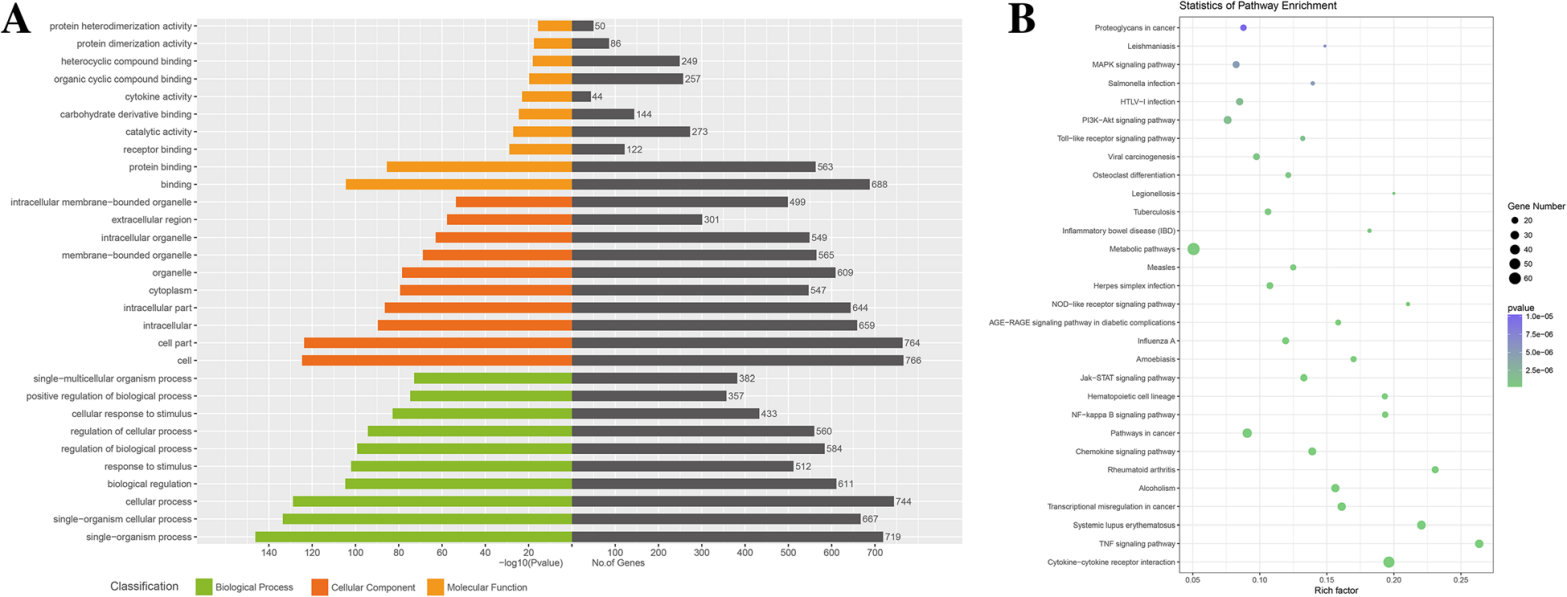
GO and KEGG analysis. **a** Top 10 terms from a GO analysis of molecular function, biological process and cellular component. **b** DE mRNAs were clustered by KEGG analysis, and the top 30 pathways are shown

We performed GO analysis on the DE mRNAs and lncRNAs. The top 10 GO terms related to biological processes, cellular components and molecular function are shown in Fig. 4a and Table 3. In the biological process domain, the top 5 GO terms associated with DE mRNAs were single-organism process, single-organism cellular process, cellular process, biological regulation and response to stimulus. In the cellular component domain, the top 5 GO terms associated with DE mRNAs were cell, cell part, intracellular, intracellular part and cytoplasm. In the molecular function domain, the top 5 GO terms associated with DE mRNAs were binding, protein binding, receptor binding, catalytic activity and carbohydrate derivative binding. KEGG analysis of the DE mRNAs determined that 122 pathways were significantly altered in MSCs co-cultured with CD14^+^ monocytes. The top 30 affected pathways are shown in Fig. 4b. The top 10 pathways and DE mRNAs associated with these pathways are shown in Table 4. The top pathways included cytokine-cytokine receptor interactions, TNF signalling pathway, chemokine signalling pathway and NF-kappa B signalling pathway, which contribute to the immunoregulatory function of MSCs, were identified.

**Table 3:** GO analysis of DE mRNA

Term	Domain	Count	*P* value	Corrected *P* value
Single-organism process	Biological process	719	5.62E−147	3.50E−143
Single-organism cellular process	Biological process	667	2.68E−134	8.34E−131
Cellular process	Biological process	744	1.41E−129	2.93E−126
Biological regulation	Biological process	611	2.08E−105	3.24E−102
Response to stimulus	Biological process	512	9.72E−103	1.21E−99
Regulation of biological process	Biological process	584	6.30E−100	6.54E−97
Regulation of cellular process	Biological process	560	5.33E−95	4.74E−92
Cellular response to stimulus	Biological process	433	1.45E−83	1.13E−80
Positive regulation of biological process	Biological process	357	1.96E−75	1.35E−72
Single-multicellular organism process	Biological process	382	1.30E−73	8.13E−71
Cell	Cellular component	766	2.11E−125	1.22E−122
Cell part	Cellular component	764	1.94E−124	5.61E−122
Intracellular	Cellular component	659	2.32E−90	4.48E−88
Intracellular part	Cellular component	644	3.61E−87	5.23E−85
Cytoplasm	Cellular component	547	4.42E−80	5.11E−78
Organelle	Cellular component	609	3.54E−79	3.41E−77
Membrane-bounded organelle	Cellular component	565	1.51E−69	1.25E−67
Intracellular organelle	Cellular component	549	1.17E−63	8.48E−62
Extracellular region	Cellular component	301	1.98E−58	1.27E−56
Intracellular membrane-bounded organelle	Cellular component	499	2.71E−54	1.57E−52
Binding	Molecular function	688	3.91E−105	4.12E−102
Protein binding	Molecular function	563	3.38E−86	1.78E−83
Receptor binding	Molecular function	122	1.25E−29	4.38E−27
Catalytic activity	Molecular function	273	7.99E−28	2.10E−25
Carbohydrate derivative binding	Molecular function	144	2.98E−25	6.27E−23
Cytokine activity	Molecular function	44	9.25E−24	1.62E−21
Organic cyclic compound binding	Molecular function	257	1.80E−20	2.70E−18
Heterocyclic compound binding	Molecular function	249	8.28E−19	1.09E−16
Protein dimerization activity	Molecular function	86	3.02E−18	3.53E−16
Protein heterodimerization activity	Molecular function	50	2.05E−16	2.16E−14

**Table 4:** Pathways with the largest significant difference in KEGG analysis

Pathway	Count	*P* value	Corrected *P* value	Gene
Cytokine-cytokine receptor interaction	52	1.14E−27	2.92E−25	IL15RA, IL6, IL11, CCR7, TNFRSF9, IL15, KIT, CXCL10, INHBA, TNFRSF21, CSF3, CCL8, IL26, IL23A, CXCL12, IL11RA, CXCL3, TNFRSF8, CCL20, CCL2, PF4V1, LIF, TNFSF10, IL1R2, CCL3, BMP2, IL20RB, IL1B, IL1A, IL1R1, CXCL8, IL7R, RELT, CXCL1, VEGFA, GDF5, IFNGR1, IL18RAP, CSF2, CXCL6, TNFSF11, IL18R1, TNFRSF1B, CXCL5, CCL7, TGFBR1, PRL, IL24, CXCL2, CCL5, CXCL11, OSMR
TNF signalling pathway	29	5.46E−19	7.02E−17	IL6, ICAM1, LIF, CXCL10, MMP3, BIRC3, TNFAIP3, CXCL3, CXCL2, CCL20, IL15, CEBPB, CCL2, NOD2, IL1B, NFKBIA, CXCL1, MAP3K8, JAG1, CSF2, EDN1, IL18R1, TNFRSF1B, CXCL5, MAP3K5, CCL5, PTGS2, SOCS3, MAP3K14
Systemic lupus erythaematosus	30	9.58E−18	8.21E−16	HIST1H3B, HIST1H4A, HIST1H2AH, HIST1H4I, HIST1H3C, HIST1H3J, HIST1H3G, HIST1H2AK, HIST1H2AJ, HIST1H2AL, HIST1H2AG, HIST3H2BB, FCGR3A, HIST1H4L, HIST2H2BF, HIST1H2BE, HIST1H2BN, HIST1H2BL, HIST1H2BM, C3, HIST1H2AI, HIST2H3D, C8A, C1S, HIST2H3A, HIST1H2BJ, HIST2H3C, HIST1H2AE, HIST1H2AD, HIST1H2BB
Transcriptional misregulation in cancer	29	4.51E−14	2.90E−12	IL6, HIST1H3B, CCR7, BCL2A1, NFKBIZ, HIST1H3J, NR4A3, HIST1H3G, CD14, BIRC3, HIST2H3D, CDKN2C, IGF1, CEBPB, HIST1H3C, IL1R2, PLAU, GRIA3, CXCL8, ID2, HIST2H3A, CSF2, MEF2C, ETV1, MMP3, HIST2H3C, SPINT1, MEIS1, FOXO1
Alcoholism	28	2.35E−13	1.21E−11	HIST1H3B, HIST1H4A, HIST1H4I, HIST1H3C, HIST1H3J, HIST1H3G, HIST2H3A, HIST1H2AK, HIST1H2AJ, HIST1H2AL, MAOA, HIST1H2AG, HIST3H2BB, HIST2H2BF, HIST1H2BE, HIST1H2BN, HIST1H2BL, HIST1H2BM, HIST1H2AI, HIST2H3D, HIST1H2AH, HIST1H4L, SLC29A1, HIST1H2BJ, HIST2H3C, HIST1H2AE, HIST1H2AD, HIST1H2BB
Rheumatoid arthritis	21	3.59E−13	1.54E−11	IL6, IL1B, IL1A, CXCL12, CSF2, CXCL6, CCL20, IL11, IL15, CXCL8, CXCL5, CCL2, TLR2, CXCL1, ICAM1, MMP3, CCL5, VEGFA, IL23A, TNFSF11, CCL3
Chemokine signalling pathway	26	1.79E−11	6.59E−10	CCR7, GRK3, ITK, CXCL10, CXCL12, CXCL3, CXCL2, CCL20, TIAM1, CCL2, PF4V1, CCL3, JAK3, PLCB4, JAK2, NFKBIA, CXCL8, CXCL1, STAT1, CXCL6, CXCL11, CXCL5, CCL7, CCL8, CCL5, HCK
Pathways in cancer	36	2.09E−10	6.08E−09	IL6, FGF11, KIT, NKX3–1, EGLN3, BIRC5, MITF, BIRC3, BDKRB1, WNT5A, IGF1, CXCL12, FGF7, ABL1, PTGS2, WNT5B, FGF5, EDNRB, RAD51, PLD1, BMP4, PLCB4, NFKBIA, CXCL8, NFKB2, AR, VEGFA, BMP2, FZD3, COL4A4, STAT1, SLC2A1, FGF2, LPAR1, TGFBR1, FOXO1
NF-kappa B signalling pathway	18	2.13E−10	6.08E−09	IL1B, CD14, CXCL12, IL1R1, TNFAIP3, CXCL2, BCL2A1, PTGS2, ICAM1, NFKBIA, CXCL8, NFKB2, RELB, LBP, PLAU, BIRC3, TNFSF11, MAP3K14
Haematopoietic cell lineage	17	6.91E−10	1.78E−08	IL6, IL1B, IL1A, IL1R1, ITGA1, CSF2, CD1D, IL11, CD38, ITGA4, IL7R, CD55, CSF3, KIT, CD14, IL1R2, IL11RA

### Interaction and co-expression network analysis

**Fig. 5:**
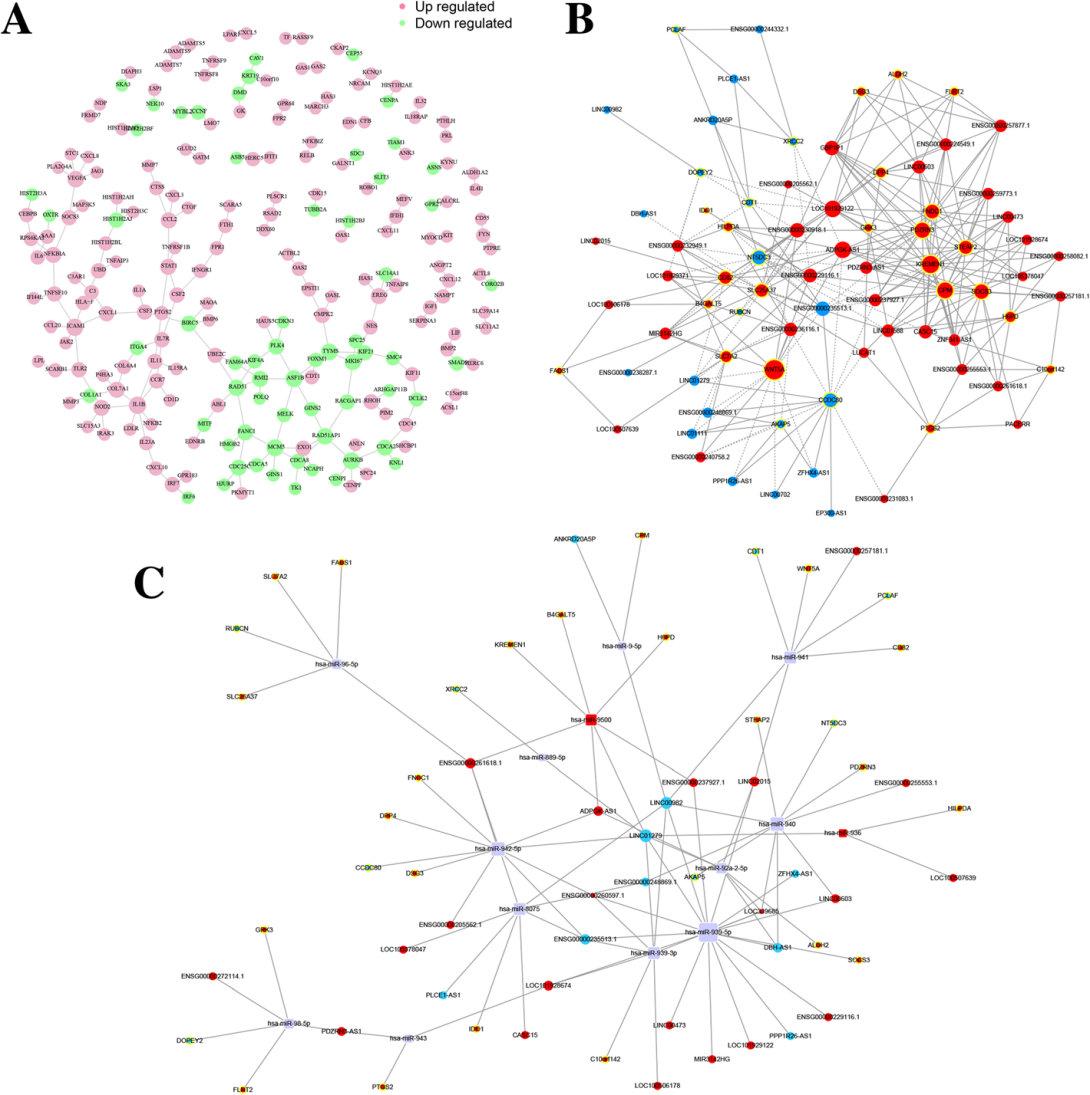
Interaction and co-expression network analysis. **a** Interactions between DE mRNAs. Purple indicates upregulated genes, and green indicates downregulated genes. **b** Co-expression network of DE lncRNAs and DE mRNAs. **c** CeRNA network between mRNAs, lncRNAs and predicted miRNAs

The interactions between proteins coded by DE mRNAs are shown in Fig. 5a. CCL2, IL1B, COL7A1 and ICAM1 are the key genes that interacted with many other DE mRNAs in this network. In addition, a co-expression network was constructed for DE lncRNAs and mRNAs (Fig. 5b). Within these RNA networks, LOC101929122 has the maximum number of targets, including 28 DE mRNAs, and WNT5A has the maximum number of co-expressed lncRNAs. The top 10 co-expression pairs are shown in Table 5. lncRNAs act as ceRNAs to exert their biological function [27]; thus, we constructed a ceRNA network from all of the DE mRNAs and lncRNAs in order to identify possible regulatory miRNAs (Fig. 5c). MiR-939-5p, miR-940 and miR-8075 were enriched in the ceRNA network, indicating that they play critical roles in the DE mRNA-lncRNA functional network and in the immunoregulatory function of MSCs.

**Table 5:** The top 10 co-expression pairs

mRNA	Gene	lncRNA	Gene	Correlation coefficient	*Z*-score	*P* value
NM_001346144.1	FLRT2	NR_104633.1	LINC00603	0.991686	2.981175	0.002871
NM_001944.2	DSG3	ENST00000551450.1	ENSG00000257877.1	0.956165	2.964272	0.003034
NM_001008539.3	SLC7A2	ENST00000413096.1	ENSG00000236116.1	0.980629	2.927594	0.003416
NM_032532.2	FNDC1	NR_003133.2	GBP1P1	0.981098	2.911009	0.003603
NM_001346144.1	FLRT2	ENST00000551450.1	ENSG00000257877.1	0.956947	2.900753	0.003723
NM_032532.2	FNDC1	ENST00000558317.1	ENSG00000259773.1	0.952101	2.899614	0.003736
NM_001039570.2	KREMEN1	ENST00000558317.1	ENSG00000259773.1	0.957745	2.898683	0.003747
NM_001039570.2	KREMEN1	NR_003133.2	GBP1P1	0.95919	2.895504	0.003786
NM_000690.3	ALDH2	ENST00000551450.1	ENSG00000257877.1	0.963804	2.889299	0.003861
NM_001935.3	DPP4	NR_121668.1	LOC101929122	0.952931	2.885277	0.003911

### Analysis of lncRNA target mRNAs

**Fig. 6:**
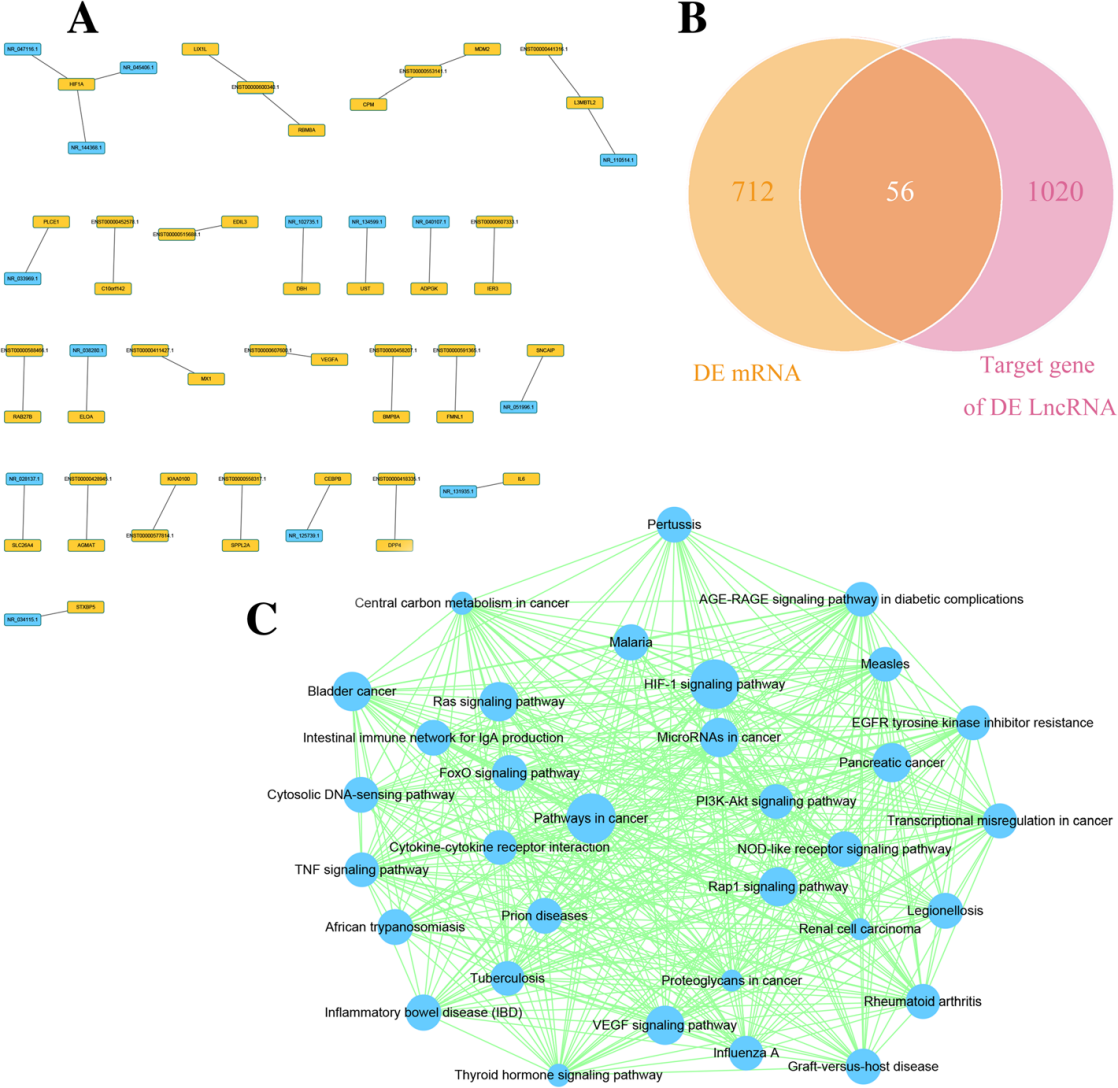
Analysis of lncRNA target mRNAs. **a** DE lncRNAs and their target genes with a combined score larger than 0.9 are shown. Yellow indicates upregulated genes, and blue indicates downregulated genes. **b** Venn diagram showing DE mRNAs and target genes of DE lncRNAs. **c** Target genes of DE lncRNAs were clustered using KEGG pathway analysis

Next, we analysed the possible target genes of the DE lncRNAs. A total of 1076 target genes of these DE lncRNAs were identified. Target genes with a combined score higher than 0.9 are shown in Fig. 6a. Figure 6a shows that HIF1A was the target gene of three DE lncRNAs, including NR_047116.1, NR_045406.1 and NR_144368.1. Moreover, IL6, an important cytokine, was the target of the DE lncRNA NR_131935.1. A Venn diagram analysis demonstrated that 56 mRNAs were found at the intersection of the 768 DE mRNAs and the 1076 DE lncRNA target genes (Fig. 6b). A list of these 56 mRNAs is shown in Additional file 3: Table S3. The list includes several known inflammatory-related molecules, such as IL6, SOCS3 and IDO1. We also performed KEGG pathway analysis on the DE lncRNA target genes to identify pathway clusters. The protein-protein interactions between these targets and the relationships between these pathways are shown in Fig. 6c, including the TNF signalling pathway, cytokine-cytokine receptor interactions and the HIF-1 signalling pathway.

## Discussion

In our present research, we utilized high-throughput sequencing followed by bioinformatic analysis to analyse the mRNA and lncRNA expression profiles and functional networks of MSCs co-cultured with CD14^+^ monocytes. These findings were then confirmed by qPCR. KEGG pathway analysis indicated that some key pathways, such as cytokine-cytokine receptor interactions, the TNF signalling pathway, the chemokine signalling pathway and the NF-kappa B signalling pathway, contribute to the immunoregulatory function of MSCs in monocytes. Other bioinformatic analyses revealed interactions between mRNAs, lncRNAs and miRNAs. Additionally, we identified target genes of the DE lncRNAs as well as the intersection of these genes with DE mRNAs and performed KEGG pathway analysis on the DE genes. Our results provide a model that can be used to explore the roles of lncRNAs and mRNAs in MSC-mediated immunoregulatory mechanisms.

The homeostasis of the immune system depends on the balance between immunocytes and their regulatory cells [18]. MSCs are one of the most important immunoregulatory cell types [13, 28]. MSCs are pluripotent stem cells that regulate the functions of many immune cells, including T lymphocytes, B lymphocytes and dendritic cells [4, 19, 29]. Previous studies have demonstrated that MSCs can affect monocytes and macrophages in a paracrine manner in a co-culture system. For example, Ko et al. [30] found that MSC-primed monocytes/macrophages express high levels of MHC class II, B220, CD11b and IL-10 and exhibit T cell–suppressive activities in a TNF-α–stimulated gene/protein (TSG)-6–dependent manner. Similar findings are described in Melief et al. [31]. This group showed that MSCs can promote monocyte survival and induce differentiation towards macrophage type 2 cells, which express CD206 and CD163 and secrete high levels of IL-10 and CCL-18. This powerful immunoregulatory ability allows MSCs to play wide-ranging roles in maintaining the dynamic equilibrium of the immune system in vivo. Monocytes are critical constituents of the immune system [18]. Monocytes are active immunocytes that migrate to specific tissues and then differentiate into macrophages and dendritic cells upon stimulation by various factors [15]. MSCs play an essential role in accelerating monocyte migration and inhibiting M1 macrophage differentiation; these observations were confirmed in this study [32–34]. On the one hand, MSCs exhibit a considerable therapeutic effect in several diseases through the secretion of cytokines that modulate monocyte function [9, 28, 35]. On the other hand, abnormal immunoregulation by MSCs could lead to several autoimmune diseases [11–13]. Therefore, it is important to elucidate a detailed mechanism of MSC-mediated immunoregulation of monocytes in order to improve their curative effects and to illuminate the pathogenesis of autoimmune diseases.

mRNA expression profiles reflect the biological behaviours and functions of cells. mRNA expression profiles are altered when cells are co-cultured with other cells. These changes in mRNA expression profiles are closely related to their functions in other cells [36]. To explore the mechanism of MSC functions in monocytes, we analysed the mRNA profile by gene sequencing and bioinformatics analysis. This analysis identified 768 DE mRNAs. Among these DE mRNAs, CCL8, CXCL6, CCL20, CXCL5, CXCL8, CXCL3 and CXCL10 were the most significantly upregulated. These cytokines play important roles in the migration and polarization of monocytes [37–40]. These results indicate that MSCs affect monocyte migration and polarization mainly through these key cytokines. In addition, GO analysis demonstrated enrichment of specific molecular functions, such as cytokine activity, indicating the critical role of these cytokines in the immunoregulatory ability of MSCs. KEGG analysis identified 122 signalling pathways associated with the DE mRNAs. Cytokine-cytokine receptor interactions, the TNF signalling pathway, the chemokine signalling pathway and the NF-kappa B signalling pathway were the pathways with the largest significant differences. In a previous study, NF-kappa B-mediated activation of MSCs led to the secretion of TNF-α and other cytokines, which was related to MSC-based therapeutic efficacy [41]. Therefore, we suggest that the NF-kappa B signalling pathway is activated in MSCs co-cultured with monocytes. This activation leads to the secretion of cytokines, including TNF-α, CCL and CXCL, which ultimately regulate the migration and polarization of monocytes. Notably, the SLE and rheumatoid arthritis pathways were also among the pathways identified as having the largest significant difference by KEGG analysis. These results are consistent with those of a previous study and emphasize the importance of MSC-mediated immunoregulation of monocytes in the pathogenesis and clinical treatment of autoimmune diseases [42, 43].

mRNA expression profiles are under the control of a series of epigenetic regulators, of which lncRNAs are an important component [21]. A lncRNA is a transcript longer than 200 nt that does not have protein coding ability but exerts indispensable regulatory functions [44]. Numerous studies have confirmed that lncRNAs regulate the differentiation of MSCs [45, 46]. For example, the lncRNA GAS5 negatively regulates MSC adipogenesis through the miR-18a/CTGF axis [25]. However, the specific lncRNAs involved in the immunoregulatory capacity of MSCs remain unknown. In this study, we first detected lncRNA expression profiles involved in MSC immunoregulatory functions. We found that 145 lncRNAs were DE in MSCs upon co-culture with monocytes. These DE lncRNAs may contribute to the differential expression of mRNAs and thus may be involved in the immunoregulatory function of MSCs. One of the most critical roles of lncRNAs is their ability to act as ceRNAs for miRNAs [27]. In this study, we constructed a ceRNA network between DE mRNAs and lncRNAs, which allowed us to identify several key miRNAs, including miR-939-5p, miR-940 and miR-8075. MiR-940 promotes the osteogenic differentiation of MSCs [47]. Additionally, exosomal miR-940 induces M2 polarization of monocytes [48]. Here, we suggest that a DE lncRNA-miR-940-mRNA network exists in MSCs and plays an active role in their immunoregulatory function.

To further study the mechanism of these lncRNAs, we identified 1076 target mRNAs of the DE lncRNAs. KEGG analysis identified a total of 33 enriched pathways, including the TNF signalling pathway and cytokine-cytokine receptor interaction pathway, further emphasizing their importance in our study. However, it is more important to determine whether these targets overlap with DE mRNAs. Using a Venn diagram analysis, we identified 56 genes that were included in both the DE mRNA cluster and the target mRNA of the DE lncRNA cluster. One of these 56 genes was IL6, which is a cytokine that participates in the immunoregulatory function of MSCs [49]. Our results indicate that DE lncRNA NR_131935.1 and its target gene, DE mRNA IL6 (shown in Fig. 6a), could comprise another functional axis important for MSC-mediated immunoregulation of monocytes.

Ankylosing spondylitis (AS) is an autoimmune disease characterized by chronic inflammation and pathological osteogenesis [50]. Abnormal MSC immunoregulation contributes to the pathogenesis of autoimmune diseases [51, 52]. Recently, we demonstrated that CCL2 is overexpressed in MSCs from AS patients, inducing the monocyte disorder and eventually leading to the chronic inflammation characteristic of AS [53]. However, the mechanism and underlying cause of CCL2 overexpression remain unclear. CCL2, also called MCP1, is a secreted protein involved in immunoregulatory and inflammatory processes [54]. In the present study, MSC CCL2 expression was significantly upregulated upon co-culture with monocytes, confirming that CCL2 is involved in MSC-mediated immunoregulation of monocytes, as previously reported [55]. In addition, SOCS3, a regulator of CCL2 expression, was identified within the DE mRNA cluster and is the target gene of a DE lncRNA, LOC101928674. Therefore, we suggest that a LOC101928674-SOCS3-CCL2 axis may exist and may be involved in MSC-mediated immunoregulation of monocytes. We further suggest that abnormal LOC101928674 expression may contribute to CCL2 overexpression in MSCs from AS patients and that a LOC101928674-SOCS3-CCL2 axis may be involved in the pathogenesis of AS. However, further research should be performed to confirm this hypothesis.

In summary, our research presents lncRNA and mRNA expression profiles and functional networks of MSCs involved in the regulation of monocytes. Several candidate mechanisms were identified, including the LOC101928674-SOCS3-CCL2 axis. Our results provide possible molecular mechanisms for the immunoregulatory function of MSCs, which may help to elucidate the pathogenesis of autoimmune diseases and improve the clinical use of MSCs. However, the specific lncRNAs that function in MSCs and their associated mechanisms are still unknown. Further studies should address these questions.

## Conclusions

Our research describes the MSC lncRNA and mRNA expression profiles and functional networks involved in the regulation of monocytes. Our results provide possible molecular mechanisms for the immunoregulatory function of MSCs. Several candidate mechanisms were discussed, including the LOC101928674-SOCS3-CCL2 axis. These proposed mechanisms may help to elucidate possible molecular therapeutic targets for MSC-based autoimmune diseases.

## Additional files

Additional file 1: **Table S1.** Donor information. (DOCX 14 kb)

Additional file 2: **Table S2.** Primers used for qRT-PCR. (DOCX 17 kb)

Additional file 3: **Table S3.** The intersection of DE mRNAs and DE lncRNA target genes. (DOCX 13 kb)

Additional file 4: **Figure S1.** The proportion of M2 macrophages in 0 day and 5 days after spontaneous differentiation and the flow diagram of TNFα and IL1β by MSCs when co-culture or not with monocytes. (JPG 309 kb)

## Data Availability

Not applicable.

## Abbreviations

AS: Ankylosing spondylitis
DE: Differentially expressed
DMEM: Dulbecco’s modified Eagle medium
FBS: Foetal bovine serum
GVDH: Graft-versus-host disease
lncRNA: Long non-coding RNA
MRE: MicroRNA recognition elements
mRNA: Messenger RNA
MSC: Mesenchymal stromal cell
NLP: Natural language processing
PBMC: Peripheral blood mononuclear cells
PBS: Phosphate-buffered saline
qRT-PCR: Real-time quantitative reverse transcription-polymerase chain reaction
SLE: Systemic lupus erythaematosus
