# Supplementary material for: lncRNA-mRNA expression profiles and functional networks of mesenchymal stromal cells involved in monocyte regulation
Source: Stem Cell Res Ther. 2019 Jul 16;10:207. doi: 10.1186/s13287-019-1306-x (PMC6636070; doi:10.1186/s13287-019-1306-x)
Supplement: Supplementary file 1 — Table S1. Donor information. (DOCX 14 kb) [file 13287_2019_1306_MOESM1_ESM.docx]

|  | **Healthy donors** |
| --- | --- |
| Number | 18 |
| Age, year | 27.6±5.7 |
| No.(%) male | 10(64%) |
| No.(%) of syphilis infection | 0 |
| No.(%) of gonorrhea | 0 |
| No.(%) of HIV infection | 0 |
| No.(%) of hepatitis virus infection | 0 |

**Table S1. The donors’ information**
