# Supplementary material for: lncRNA-mRNA expression profiles and functional networks of mesenchymal stromal cells involved in monocyte regulation
Source: Stem Cell Res Ther. 2019 Jul 16;10:207. doi: 10.1186/s13287-019-1306-x (PMC6636070; doi:10.1186/s13287-019-1306-x)
Supplement: Supplementary file 2 — Table S2. Primers used for qRT-PCR. (DOCX 17 kb) [file 13287_2019_1306_MOESM2_ESM.docx]

**Table S2 Primers used for qRT-PCR**

| **Gene** | **Forward primer**  **(5`-3`)** | **Reverse primer**  **(5`-3`)** |
| --- | --- | --- |
| LINC00473 | AAAGGAGCGCTCTATGGGCT | TGGAACCGCCTGGAAACAAC |
| MIR3142HG | CCACTTCCGCCCTTTGCTTA | CCGATCTCTGGTGTCGGTTG |
| ENSG00000236116.1 | GCCAGCTGCTTTTCCATCTCT | TCGGGAATTTCCATGAAAGCAG |
| LOC339685 | TGAGAACACCAACCCACACC | GCTTCTGTCCACGAAGGGAA |
| GBP1P1 | GCTGGAAGAGCAAGAGAGGAC | TTCTCTCTGCCACAGTAAGCC |
| LINC01588 | ACAAACCAAAACACGCCAGT | TCACCCAGGACATCACAAGC |
| ENSG00000231083.1 | CAAGGACTGTGCCTGAAGGA | TCGTGAACTCCTCAACCAGG |
| ENSG00000257181.1 | CGGCTCTCATCCCCAACTATC | TCTTGGCCTGGGTTACATGG |
| ENSG00000261618.1 | TACATGGTGCTGTCTCTCAGC | ACTTCAAAGCCCATTGTTGCC |
| ADPGK-AS1 | CCAACTCCTTTCCTAGCCCG | TGTGACGTAGCGCTTGTGT |
| ENSG00000237927.1 | GAGGGTCAAAGGGCAGACAG | TCACCTCTCCTCCGTTGGTT |
| ENSG00000232949.1 | TGCCCACTACCTCATTCCCA | TGGAGTGGAGACTGTTCCGT |
| LOC541472 | GCATAACATTTCAGGACCCGC | GGAGCAGTGGCTTCGTTTCA |
| PDZRN3-AS1 | CTGGCTGTGGGTTCCCTTTA | AGACCAGCTAGGGGATCTCG |
| ENSG00000259773.1 | TGTGCCATTTCCAGACGCAT | AGTTGTTGGCAAGCCATAACATT |
| ENSG00000258082.1 | TCACGCCTCAACTTCCTCTG | AAGCAAAAGCTGCCATCGTG |
| ENSG00000240758.2 | GCCTTTCAGAAGGAGAAGGCA | TGAGGCAGGGTCCATAGACTT |
| LINC02015 | CATGTTCCCCACAGGATGTGT | TCCCGGCTGTTAAATGTGTTCT |
| ZNF341-AS1 | TTTCTGGGCTGCTGTGACTT | CGTATCGACTGGGTTGATGGT |
| ENSG00000205562.1 | TGGCAGCAGAGCATAGGGAT | CTTCCCTGTGGAACAGACAGC |
| ENSG00000272114.1 | ATCCACAGTGATTTGGGGAAGTA | TGCTTGCCATTCCCCACTTG |
| LOC100506178 | GTGGCTGGTGACTGTTCTCA | TAGCACATCGGAGGGACTCA |
| LUCAT1 | GGATGAGACTTAGCGTGCCT | CCTCGGGTTGCCTCTGTTTA |
| ENSG00000230918.1 | CAAGCCGAGCAGATCAAGTC | AAAGACTATGAGAGGCCGCA |
| ENSG00000248869.1 | CCGGGGAAATGAGGGCATAG | GGGGAAGGCAGGAGACCTTA |
| PLCE1-AS1 | TCATTATTGGCAGGTCGGGG | CGATTGTGTTAAACATCAGGGGT |
| ZFHX4-AS1 | GCCCCTGCCAACATCATTCA | AAAAGGGGAGATGTGAGGGC |
| ENST00000430320.3 | AAACGACCGACACAACCAGG | AGGCACGTCACTGGTACTTT |
| LINC01111 | TGACCAGAAGACCTGAACAGC | GGCCTTTTCAGGGACTAGCTT |
| PPP1R26-AS1 | TGAGACCTTGATTTGCGGGG | GCCGTTAGGCTGGAAAGTGA |
| ENSG00000235513.1 | ACCAAACAGCTCCAAGTCGT | TGAGGGGGCTGATAGCAGAT |
| ENSG00000260597.1 | GGAGGGGTTTTCCGCTACTC | AAAGCTGCACACCTCTTGGT |
| LINC01279 | GCCCGAGAGTAGGGTGAATG | GGGAGCGAAACGAACAGAGA |
| ENSG00000244332.1 | TTCCACAGTTTCCAGTTTGAGGA | ATGCTTGCAGGGCCAATCT |
| ENSG00000255553.1 | GCTTCACGTCACACTACAGC | CAGGAGGGATTGGCTGAACA |
| ENSG00000257877.1 | GCCACCTTGTGGGACATACA | GCAACATCGTTGGAAGGACAC |
| LINC00603 | TGAGGACCACCATCAATTCCAA | GAGTTAAGTTTCCACGCTGGC |
| LOC101928674 | CTGCTCTGCCCTCCTGATTT | TGCCTGGAAATGGGATTGTGA |
| LOC101929371 | TTGAGATGTCGAGAGCGAGC | CTTGGGCTGTGCTGAGACTA |
| ENSG00000224549.1 | GTGAAGCTCCATATGTCCCACA | GCACCTCTACTTTCACCATGAGA |
| ENSG00000244586.1 | AAAACGCACAAGTCGCCATC | CCGCACAGCAATAAGTTCCG |
| PACERR | CCTTCACCCCCTCCTTGTTT | AGCGTCCCTGCAAATTCTGG |
| CASC15 | CTCAGCCAGTGCAACACAAC | TTTTGTGGCAGGTAGGGGAC |
| LOC101929122 | GCGCTATGTCAGTTCTCCCT | CTGGCTACTGCACTGGTGTT |
| LOC105378047 | CAGCTCCAGGGAAGAACACAT | AGTTTAGTTGTGGGGGTGGTC |
| ENSG00000238287.1 | AGGGTGTGCTTATGCGAGAG | TTGACACTCCCTCATGCTCG |
| LINC00702 | CAGAAGACGAAGTGCTCCTGA | GTGAGCTGGCTTCATTCACC |
| DBH-AS1 | CTTCAGTACGTCGCGGTTGA | CTGACGGGTCTCCTCCAACT |
| ENSG00000236116.1 | AAAGCAAGCCAAGAGATGCTA | CACCTTGGGTTCCGGCTTA |
| ENSG00000240758.2 | AAGGAGAAGGCAGCTCTGTTT | AGTTGCATCGGCCACTATACA |
| ENSG00000257181.1 | TCGGCTCTCATCCCCAACTA | TGGGTTACATGGTTCCCAGC |
| LINC00982 | AGTCGAGACACGGCTCAAAG | CCATCACCTTCGGGTCACTC |
| EP300-AS1 | CCCTCTAGGGTGGAAGGCT | GTTAGGAGGTGGACCTCAGC |
| ENSG00000229116.1 | CGTCCTCGGGACCTTTCTC | CCGGGTCACAGGCTTGTATTTC |
| GAPDH | TTGAGGTCAATGAAGGGGTC | GAAGGTGAAGGTCGGAGTCA |
| CXCL2 | TGTGACGGCAGGGAAATGTA | TGCTCTAACACAGAGGGAAACA |
| CXCL3 | CCAAACCGAAGTCATAGCCAC | TGCTCCCCTTGTTCAGTATCT |
| CXCL6 | AGAGCTGCGTTGCACTTGTT | GCAGTTTACCAATCGTTTTGGGG |
| CXCL8 | ACTGAGAGTGATTGAGAGTGGAC | AACCCTCTGCACCCAGTTTTC |
| IL-6 | ACTCACCTCTTCAGAACGAATTG | CCATCTTTGGAAGGTTCAGGTTG |
| CCL2 | CAGCCAGATGCAATCAATGCC | TGGAATCCTGAACCCACTTCT |
| MMP3 | CTGGACTCCGACACTCTGGA | CAGGAAAGGTTCTGAAGTGACC |
| CFB | GCACTGGAGTACGTGTGTCC | CCCGTTCTCGAAGTCGTGTG |
| LIF | CCAACGTGACGGACTTCCC | TACACGACTATGCGGTACAGC |
| TNFAIP6 | TTTCTCTTGCTATGGGAAGACAC | GAGCTTGTATTTGCCAGACCG |
| SOSC3 | CCTGCGCCTCAAGACCTTC | GTCACTGCGCTCCAGTAGAA |
| GREM2 | ATCCCCTCGCCTTACAAGGA | TCTTGCACCAGTCACTCTTGA |
| DKK1 | CCTTGAACTCGGTTCTCAATTCC | CAATGGTCTGGTACTTATTCCCG |
| OSR1 | CGGTGCCTATCCACCCTTC | GCAACGCGCTGAAACCATA |
| CTGF | CAGCATGGACGTTCGTCTG | AACCACGGTTTGGTCCTTGG |
| DKK2 | CTCACAGATCGGCAGTTCG | ATGCCAGTCCTTGGTACATGC |
| ROR1 | CAGTCAGTGCTGAATTAGTGCC | TCATCGAGGGTCAGGTAAGAAT |
