# Supplementary material for: lncRNA-mRNA expression profiles and functional networks of mesenchymal stromal cells involved in monocyte regulation
Source: Stem Cell Res Ther. 2019 Jul 16;10:207. doi: 10.1186/s13287-019-1306-x (PMC6636070; doi:10.1186/s13287-019-1306-x)
Supplement: Supplementary file 3 — Table S3. The intersection of DE mRNAs and DE lncRNA target genes. (DOCX 13 kb) [file 13287_2019_1306_MOESM3_ESM.docx]

**Table S3 The intersection of DE mRNA and target gene of DE LncRNA**

SLC25A37, SOD2, FADS1, DPP4, TNFRSF9, ALDH2, FNDC1, CHMP1B, CD82, PDZRN3, GRK3, MAP2K6, RPS6KA6, IER3, RAB27B, CTSS, BZW1, WNT5A, DOPEY2, PTGS2, SLC44A1, NT5DC3, CPM, UACA, SOCS3, PLOD2, IL6, FMNL1, SLC7A2, IDO1, SYNE3, NAMPT, HS3ST3B1, PCLAF, KREMEN1, CDT1, CCDC80, H6PD, STEAP2, FLRT2, DSG3, KRT7, AKAP5, CCDC69, MX1, CFLAR, PRKCH, IL11, NCOA7, UHRF1, GALNT1, B4GALT5, IL1R1, C10orf142, RUBCN, XRCC2
