# Supplementary figures and images for: lncRNA-mRNA expression profiles and functional networks of mesenchymal stromal cells involved in monocyte regulation
Source: Stem Cell Res Ther. 2019 Jul 16;10:207. doi: 10.1186/s13287-019-1306-x (PMC6636070; doi:10.1186/s13287-019-1306-x)

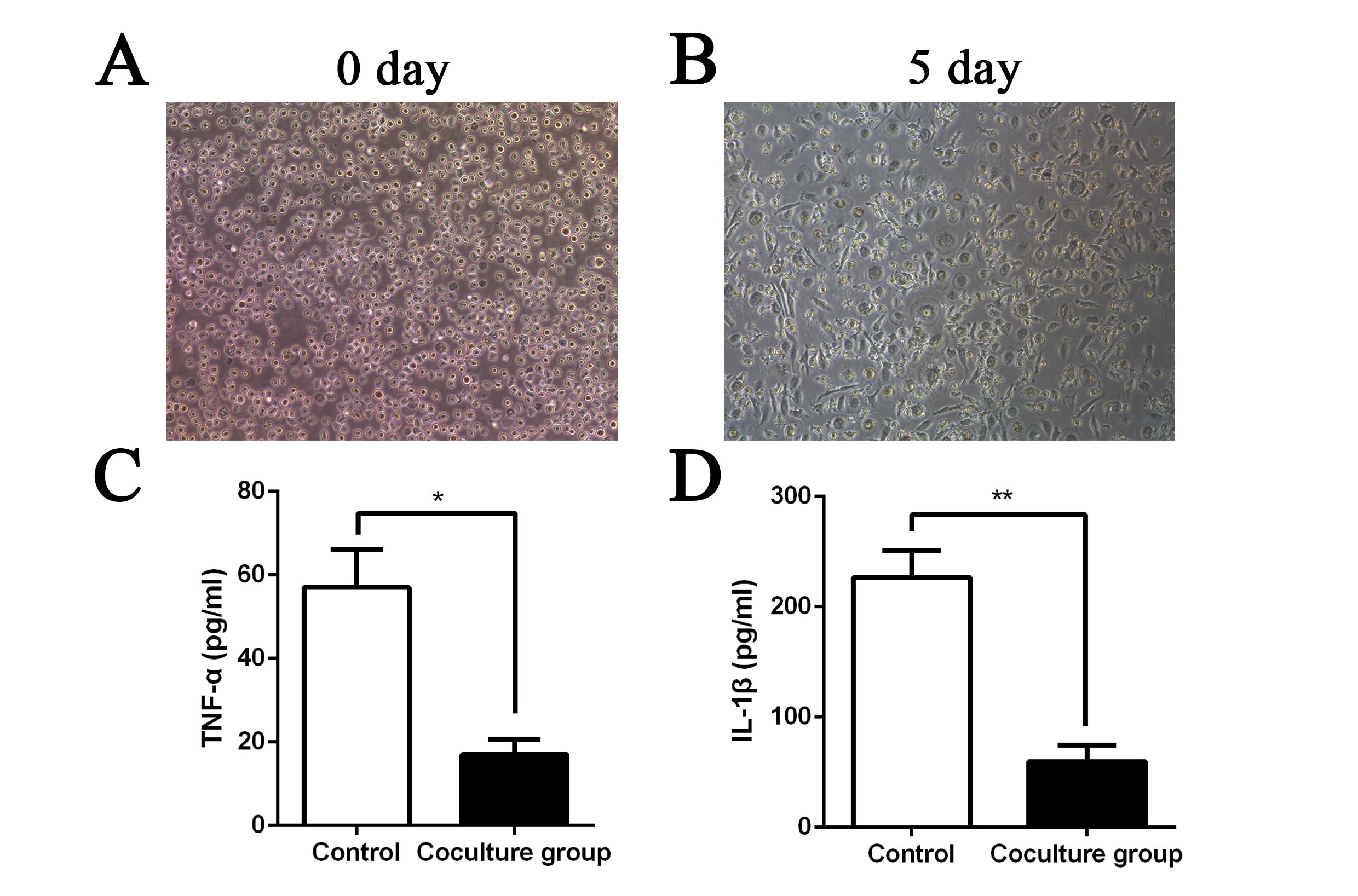

Supplement: Supplementary file 4 — Figure S1. The proportion of M2 macrophages in 0 day and 5 days after spontaneous differentiation and the flow diagram of TNFα and IL1β by MSCs when co-culture or not with monocytes. (JPG 309 kb) [file 13287_2019_1306_MOESM4_ESM.jpg]
